# The role of gut microbiota in gout: Is gut microbiota a potential target for gout treatment

**DOI:** 10.3389/fcimb.2022.1051682

**Published:** 2022-11-24

**Authors:** Shuting Tong, Peiyu Zhang, Qi Cheng, Mo Chen, Xin Chen, Zitao Wang, Xiaoyong Lu, Huaxiang Wu

**Affiliations:** Department of Rheumatology, The Second Affiliated Hospital, Zhejiang University School of Medicine, Hangzhou, China

**Keywords:** gout, hyperuricemia, gut microbiota, probiotics, prebiotics

## Abstract

Numerous studies have demonstrated that gut microbiota is essential for the host’s health because it regulates the host’s metabolism, endocrine, and immune systems. In recent years, increasing evidence has shown that gut microbiota plays a role in the onset and progression of gout. Changes in the composition and metabolism of the gut microbiota, result in abnormalities of uric acid degradation, increasing uric acid generation, releasing pro-inflammatory mediators, and intestinal barrier damage in developing gout. As a result, gout therapy that targets gut microbiota has drawn significant interest. This review summarized how the gut microbiota contributes to the pathophysiology of gout and how gout affects the gut microbiota. Additionally, this study explained how gut microbiota might serve as a unique index for the diagnosis of gout and how conventional gout treatment medicines interact with it. Finally, prospective therapeutic approaches focusing on gut microbiota for the prevention and treatment of gout were highlighted, which may represent a future avenue in gout treatment.

## 1 Introduction

Gout is a common disease characterized by the deposition of monosodium urate (MSU) crystals in joint and non-joint structures (Dalbeth et al., 2021). The inflammatory response of host tissue to deposit monosodium urate (MSU) crystals induces clinical symptoms (Dalbeth et al., 2019). Globally, gout is highly prevalent. Adults in China have a gout prevalence rate of 1.1%, compared to 3% to 4% in the United States and 1% to 4% in Europe (Dehlin et al., 2020). Genetic diversity, environmental exposure, gene-environment interaction, and intrinsic risk factors (including age, gender and weight) contribute to the risk of developing gout (Major et al., 2018). In addition, gout and hyperuricemia have many common comorbidities, such as cardiovascular disease, chronic kidney disease, diabetes, metabolic syndrome and neurodegenerative diseases (Bardin and Richette, 2017).

The human digestive system contains trillions of species, including bacteria, fungi, archaea, viruses, and protozoa, which comprise the gut microbiota, a complex ecological community (Human Microbiome Project, 2012). The taxonomic diversity of the gut microbiota impacts the integrity of the epithelial barrier, preservation of intestinal metabolism, and immunological homeostasis (Parker et al., 2020). The gut microbiota influences healthy physiological function and disease susceptibility through its collective metabolic activity and host interaction (Lozupone et al., 2012). With the advancement of sequencing technology and the creation of new bioinformatics, it has been discovered that gut microbiota composition changes and metabolism disruptions are connected to the pathogenesis of numerous diseases, such as autoimmune disease (Jiao et al., 2020), mental illness (Jarbrink-Sehgal and Andreasson, 2020), cerebrovascular disease (Xu et al., 2020), and disorders of the central nervous system (Vuotto et al., 2020).

Emerging evidence revealed a link between gut microbiota and arthritis diseases, including gout (Chu et al., 2021). Therefore, this review aims to summarize gut microbiota function in gout pathogenesis and illustrate gut microbiota as a potential target of gout treatment.

## 2 Gut microbiota and physiologic acid uric metabolism

In humans and higher primates, urate is the final oxidation product of purine catabolism (Cabau et al., 2020). It is synthesized mainly in the liver, intestines and tissues, such as muscles, kidneys, and vascular endothelium (El Ridi and Tallima, 2017). Purine synthesis begins with 5-phosphoribosyl-alpha-1-pyrophosphate (PRPP) leading to hypoxanthine nucleotide formation (Dewulf et al., 2022). Hypoxanthine is converted to xanthine, which is then transformed into uric acid (UA) by either xanthine oxidase (XO) or xanthine dehydrogenase (Sun et al., 2022). Approximately 700 mg of UA is produced daily by such processes (Yanai et al., 2021). Renal and gut excretions accounts for around two-thirds and one-third of urate excretion, respectively (Mandal and Mount, 2015). Urate homeostasis is primarily influenced by renal proximal tubule cells, which express several transporters that either reabsorb urate or are involved in urate excretion (Eckenstaler and Benndorf, 2021).

During the evolution of humanoid primates, the pseudogenization of the uricase gene caused humans and other mammals to lose uricase activity. It left them unable to oxidize further urate to the more water-soluble compound, allantoin (Chang, 2014).Therefore, serum uric acid (sUA) levels in humans are three- ten times higher than in organisms that preserve uricase (Kratzer et al., 2014).

Unlike humans, bacteria can degrade uric acid by urate oxidase (uricase), and specific bacterial strains also exhibit xanthine dehydrogenase (XDH) inhibitory activity (Alam et al., 2011). *Lactobacillus* species break down inosine and guanosine to inhibit uric acid biosynthesis during purine metabolism (Alvarez-Lario and Macarron-Vicente, 2010; Wu et al., 2020). Recently, *Lactobacillus gallinii* has been shown to reduce purine levels in the gut, and its fermentation products have urate-lowering effects (Li et al., 2014). In addition, *Lactobacillus gasseri* strains can reduce purine absorption in the gut (Yamada et al., 2016). However, not all gut microbiota is protective. Xi et al. demonstrated that *Escherichia-Shigella* secretes xanthine deaminase, converting hypoxanthine and xanthine into uric acid and elevating serum uric acid levels (Xi et al., 2000).

The gut microbiota also plays a role in uric acid excretion. Studies have shown that two short-chain fatty acids (SCFAs) (propionate and butyrate) provide adenosine triphosphate (ATP) to the intestinal wall cells and promote UA excretion (Nieuwdorp et al., 2014). In addition, a recent study found higher *Escherichia coli* levels in greater uric acid decomposition (Liu et al., 2020).

## 3 Dysbiosis and gout

### 3.1 Description of the microbiome in gout patients

The abnormal secretion of interleukin-1β (IL-1β) stimulated by MSU causes the acute onset of gout, which occurs by activating the innate immune system through the recognition of Toll-like receptor (TLR) or NOD-like receptor (NLR) (Dalbeth et al., 2021). The release of a large amount of IL-1β by activating of NLRP3 (NOD-, LRR-, and pyrin domain-containing 3 inflammasome) is the central process of MSU-mediated gout acute attack (Martinon et al., 2006).

A recent study reported that *Phascolarctobacterium* and *Bacteroides* were enriched in gout patients and identified a “core microbiota” for the gout group encompassing three *Bacteroides* species (Mendez-Salazar et al., 2021). *Bacteroides* is a gut enterotype reported to promote urate conversion into allantoin, and might be involved in serum urate level regulation in humans (Lim et al., 2014).

GUO et al. observed that the gut microbiota of gout was characterized by significantly-impaired butyric acid synthesis (Guo et al., 2016). Vieira et al. found that SCFAs were necessary to assemble inflammasome and produce IL-1β (Vieira et al., 2015). Another study explored the effects of a high-fiber diet and acetate on inflammation in gout mice models. The regression of neutrophil inflammation was found to be related to a decrease of nuclear factor κ B (NF-κ B) activity and an increase of anti-inflammatory mediators (including interleukin-10, tumor necrosis factor-β and Annexin A1). Acetate controlled the inflammatory response to the MSU lens by promoting the regression of the inflammatory response (Vieira et al., 2017). The species that make SCFAs have a protective effect on inflammation, and are more abundant in the healthy group (Sheng et al., 2021). These results collectively showed that SCFAs and gut microbiota play a role in controlling inflammatory response to MSU crystals (Cleophas et al., 2017). Additionally, by up-regulating TLR2/4/5 and promoting the release of IL-1β and tumor necrosis factor-α (TNF-α), the increase in the number of inflammation-related microbiota causes immunological diseases and intestinal barrier dysfunction. Increased intestinal permeability, positively linked with serum uric acid level, results from decreased levels of the epithelial tight junction proteins occludin and claudin-1 (Lv et al., 2020).

### 3.2 Changes of gut microbiota composition and hyperuricemia

#### 3.2.1 Diversity and abundance of gut microbiota in hyperuricemia

The variety and richness of gut microbiota have changed in hyperuricemic individuals and rats, indicating that gut microbiota may have a possible involvement in gout (Liu et al., 2020; Chu et al., 2021). Uric acid is the final product of purine metabolism and alterations in uric acid production or excretion can lead to abnormal serum uric acid levels (Balakumar et al., 2020). The changes in the abundance and composition of gut microbiota increase the serum uric acid level through the dysfunction of uric acid degradation and increased uric acid production (Sheng et al., 2021).

Gout has higher relative abundances of *Prevotella* and *Bacteroides* while lower relative abundances of *Enterobacteriaceae*, which might cause uric acid degradation dysfunction and the buildup of uric acid in gout (Chu et al., 2021). Additionally, the greater serum urate (SU) level was closely connected to the lower relative abundance of *Faecalibacterium* in hyperuricemia (Wei et al., 2021).

A shotgun metagenomic study revealed the microbiota with the allantoinase gene, which can convert uric acid into urea was deficient in gout. In contrast, the microbiota with the xanthine dehydrogenase gene was abundant. The buildup of uric acid may be caused by excessive xanthine dehydrogenase and a relative lack of allantoinase (Guo et al., 2016; Gong et al., 2020).

**Figure 1:**
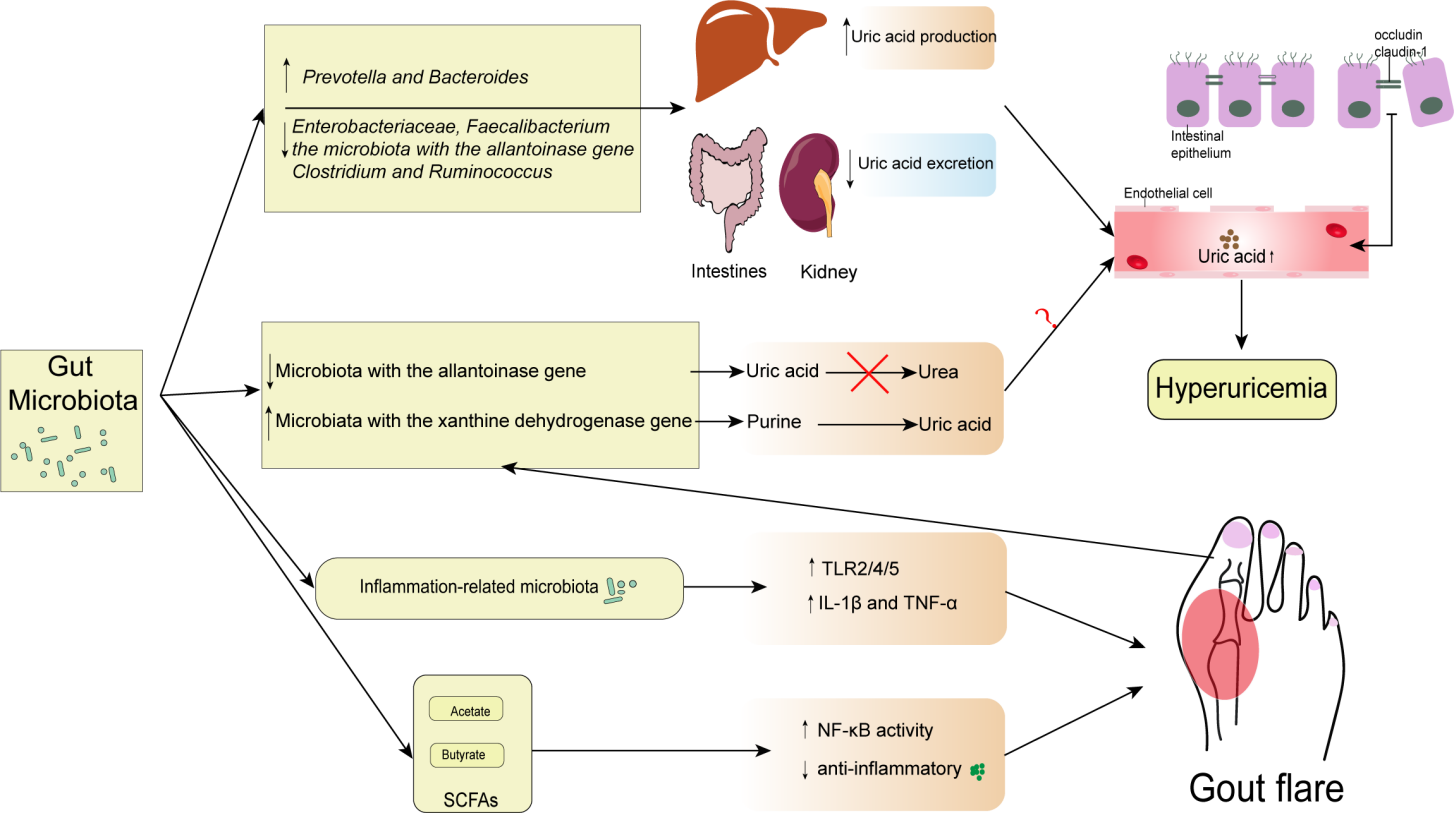
Interactions between gut microbiota and gout. The diversity and abundance of gut microbiota change include the increase of *Prevotella* and *Bacteroides* and the decrease of *Enterobacteriaceae*, *Faecalibacterium*, the microbiota with the allantoinase gene, *Clostridium*, and *Ruminococcus*. These changes result in excessive uric acid production in the liver and insufficient uric acid excretion in the kidney and intestine, raising serum uric acid levels above normal. In addition, some microbiota with the allantoinase and the xanthine dehydrogenase gene changed in gout can directly regulate the intestinal uric acid levels. However, the contribution of elevated intestinal uric acid levels to elevated serum uric acid levels remains unknown. Consequently, occludin and claudin-1 levels at tight epithelial junctions can drop when serum uric acid levels rise. Gout is caused by inflammation-related bacteria that upregulate TLR2/4/5 and encourage the release of IL-1 β and TNF- α. However, some SCFAs may have a protective role in inflammation. SCFAs, especially butyrate, are associated with the increased expression of Inhibitory-κκBα (I-κBα), which inhibits the phosphorylation and nuclear translocation of NF-κB p65, and the downstream inflammatory cytokine, MCP-1, and IL-1β expression.

An increase in UA in blood circulation affects the intestinal environment, causing changes in the gut microbiota (Wang et al., 2022). A metagenomic study reveals that hyperuricemia causes an imbalance in the gut microbiota and alters its composition. It might induce the gut microbiota to translocate into other tissues, particularly the kidney, causing inflammation (Xu et al., 2019). In addition, a recent study has demonstrated that the abundance of inflammation-related microbiota in hyperuricemia and increased uric acid levels are associated with the impairment of intestinal barrier, which disrupts the host-microbiota crosstalk (Lv et al., 2020). While luminal UA can play a protective role in intestinal injury, some studies have shown that the changes in gut microbiota caused by uric acid are beneficial to the body (Wada et al., 2022). However, a study exploring the relationship between blood uric acid levels and gut microbiota in diabetic patients, found that fluctuations in uric acid within the normal range were not associated with changes in gut microbiota (Zhang et al., 2021). Therefore, further studies are needed to explore the causal relationship between the alteration of gut microbiota and hyperuricemia (**Figure 1**).

#### 3.2.2 Metabolism of gut microbiota in hyperuricemia

Various metabolites, including SCFAs, trimethylamine, amino acid derivatives, and vitamins, are produced by the gut microbiota from dietary components, including significant amounts of micronutrients, fiber, and polyphenols (Parker et al., 2020). Acetate (C2), propionate (C3), and butyrate (C4) are the most prevalent SCFAs in the human body. SCFAs are most extensively researched (Macfarlane and Macfarlane, 2003). The human body relies heavily on SCFAs. Butyric acid protects the human gut by nourishing the mucosa, fosters the development and repair of intestinal villi, boosts intestinal immunity, encourages the growth of good bacteria, and prevents the colonization of pathogens (Louis and Flint, 2009). A study through the Kyoto Encyclopedia of Genes and Genomes (KEGG) metabolic pathway analysis revealed significant differences in amino acid metabolism (phenylalanine, tyrosine and tryptophan biosynthesis, D-glutamine and D-glutamate metabolism, and phosphate and phosphonate metabolism) and nucleotide metabolism (purine metabolism) between hyperuricemia and healthy controls. The gut microbiota’s metabolic dysfunction may influence serum uric acid levels through its impact on host metabolites (Wei et al., 2021). The production of SCFAs (concentrations of acetate, propionate, and butyrate) derived from the gut microbiota in mice positively correlates with the effectiveness of treating hyperuricemia. This finding demonstrates that some beneficial bacteria decrease in HUA mice, including bacteria that produce SCFAs, such as *Clostridium* and *Ruminococcus (* Yu et al., 2018; Xu et al., 2019; Guo et al., 2021).

## 4 Gout diagnosis based on gut microbiota

Due to the causative relationship between the gut microbiota and gout development, the gout- specific gut microbiota may be a diagnostic marker. Lin et al. made a classification model using significantly-enriched bacterial genera between healthy individuals and gout patients. The result showed a high mean area under the working curve (AUC) of up to 0.973 by the receiver operating characteristic (ROC) analysis (Lin et al., 2021). Likewise, a cohort study established a diagnostic model based on 17 kinds of gout-related bacteria and reached 88.9% accuracy (Guo et al., 2016). The metagenomic analysis of gut microbiota by Chu et al. found three genes significantly enriched in the cohort gout. The AUC of the development and validation cohort were 0.91 and 0.80, respectively (Chu et al., 2021).

Furthermore, Yang et al. verified that several bacteria, including *unclassified Enterobacteriaceae*, *Roseburia*, and *Faecalibacte-rium*, have excellent diagnostic value for asymptomatic hyperuricemia (Yang et al., 2021). Therefore, the gut microbiota imbalance characterized by gout may become a non-invasive diagnostic tool for gout and asymptomatic hyperuricemia. It has a promising target for future prevention and intervention.

## 5 Treatment of gout

### 5.1 Traditional treatment and gut microbiota changes

Non-steroidal anti-inflammatory drugs (NSAIDs), glucocorticoids, and colchicine are the first-line drugs for acute gout (Kiltz et al., 2016). International guidelines describe xanthine oxidase inhibitors and uricosuric drugs as the first- and second-line treatments, respectively, in uric acid-lowering therapy (Engel et al., 2017). Gut microbiota has become an important factor in hyperuricemia and has been shown to affect the response to disease treatment (Yu et al., 2018). A recent study found substantial alterations in the gut microbiota composition and promotion of SCFA formation, particularly acetate, in gouty arthritis patients after treatment (Park and Lee, 2022).

#### 5.1.1 Non-steroidal anti-inflammatory drugs

NSAIDs are classic drugs for treating acute gout that affect pain relief and inflammation (Bindu et al., 2020). However, NSAIDs have several side effects, including gastrointestinal damage. Studies have indicated that NSAIDs can disrupt the gut microbiota equilibrium, multiplying gram-negative bacteria and decreasing gram-positive bacteria. Subsequently, pathogens activate inflammatory pathways through TLR4 and release inflammatory cytokines (Wang et al., 2021). In addition, NSAIDs can enhance intestinal permeability, making bacteria enter the mucosa (Montalto et al., 2010), leading to further changes in gut microbiota composition.

#### 5.1.2 Colchicine

Colchicine (COL) is a traditional drug for gout that can block tubulin polymerization and prevent inflammasome activatin (Tristan Pascart, 2018). However, colchicine has potential toxicity to human health. Gastrointestinal discomfort is the most common symptom of COL toxicity, including nausea, vomiting and diarrhea (Akodad et al., 2020). Shi et al. found that acute oral COL in mice significantly affected the gastrointestinal structure and substantially changed the gut microbiota’s diversity, composition, and function. This is closely related to the down-regulation of intestinal proinflammatory cytokines and the destruction of intestinal integrity in mice. Therefore, it supports the destruction of homeostasis in the intestinal microbiome and might increase the toxic burden of COL (Shi et al., 2020). Another study in the same group identified bacterial biomarkers associated with diarrhea, indicating that the adverse reactions caused by COL were closely related to the gastric microbiological disturbance. By understanding the microbiome’s role in adverse COL reactions, the gut microbiota can be targeted, and the effectiveness of COL treatment can be increased (Shi et al., 2021).

#### 5.1.3 Allopurinol 

Allopurinol, an inhibitor of xanthine oxidase, is one of the most widely-used uric acid-lowering drugs (Mackenzie et al., 2020). Yu et al. found that the gut microbiota in the allopurinol treatment group changed compared with the control group. The treatment group had increased bifidobacterium and decreased anaerobes, which may be related to the decrease in UA. In addition, Bilophila, the only reduced genus in the allopurinol treatment group (Yu et al., 2018), has been shown to cause systemic inflammation (Feng et al., 2017).

#### 5.1.4 Benzbromarone

Benzbromarone decreases blood uric acid levels and reabsorption by blocking the dominant apical (luminal) uric acid exchanger in the human proximal tubule, URAT-1 (Azevedo et al., 2019). Similar findings were made in another study, which showed that treating with benzbromarone altered the gut microbiota in the group that received it. It led to an increase in *Bifidobacterium* and a decrease in anaerobes *Butyricimonas*. In addition, benzbromarone repaired the lipid metabolism disorder in hyperuricemia rats through gut microbiota intervention (Yu et al., 2018).

#### 5.1.5 Febuxostat

Febuxostat, a nonpurine inhibitor of xanthine oxidase, treats hyperuricemia in gout patients. It inhibits oxidized and reduced forms of xanthine oxidase, reducing uric acid formation (White et al., 2018). Lin et al. detected a restriction of gut microbiota biodiversity in untreated gout patients and febuxostat partially restored this change. Functional analysis revealed that the gut microbiome of gout patients was functionally-enriched for carbohydrate metabolism but had a lower potential for purine metabolism, which was relatively enhanced in gout patients treated with febuxostat (Lin et al., 2021). Another animal study verified that febuxostat could reshape gut microbiota dysbiosis in an animal model, regulate gut-derived metabolites, and inhibit microinflammation *in vivo (* Tu et al., 2020).

### 5.2 Treatment of gout based on gut microbiota changes

#### 5.2.1 Probiotics and prebiotics

Nowadays, the low rates of urate-lowering therapy initiation and continuation and the side effects of traditional drugs remain challenges for gout treatment. These side effects include gastrointestinal toxicity, tolerance, allopurinol hypersensitivity syndrome, nephrotoxicity, and contraindications of other common comorbidities (Khanna et al., 2012; Becker et al., 2015; Vargas-Santos and Neogi, 2017; Rai et al., 2018). About 40% of gout patients suffer from chronic kidney disease (CKD) (at least stage II) and decreased GFR (Gaffo and Saag, 2008). Non-steroidal anti-inflammatory drugs, colchicine and uricosuric medicine use also are limited (Aslam and Michet, 2017). Therefore, therapies or drugs which are safer and can intervene in gout development are greatly needed.

In recent years, a better understanding of gut microbiota in the pathogenesis of gout and applying natural products in the prevention and treatment of gout have attracted widespread attention. Natural products, probiotics, probiotics and fecal microbiota transplantation (FMT) have been widely studied by new therapeutic methods acting on gut microbiota (Wu et al., 2021; Zhao et al., 2022; Xie et al., 2022). These play a role in treating gout by inhibiting the metabolism of purine and inflammatory factors and bodies, regulating the expression of transporters, and protecting the integrity of intestinal barrier. It can also increase the abundance of intestinal bacteria related to the production of SCFA and promote SCFA production, thus inhibiting the activity of XOD in serum and liver (Ni et al., 2021).

Probiotics are “live microorganisms that, when administered adequately, confer a health benefit on the host (Hill et al., 2014). *Bifidobacterium and Lactobacillus strains* are the most widely-used probiotics in functional food and dietary supplements. However, the next generation of probiotics, such as *Faecalibacterium prausnitzii, Akkermansia muciniphila*, or the genus *Clostridium*, have shown promising results (Vallianou et al., 2020).

Some *in vitro* experiments have proven that diets containing probiotics can prevent hyperuricemia by regulating the structure and function of intestinal flora. For example, *Lactobacillus fermentans* JL-3 can regulate hyperuricemia-induced intestinal microbiota dysbiosis and effectively reduce the UA level in mice (Wu et al., 2021). DM9218, as a probiotic strain, has the potential to ameliorate fructose-induced hyperuricemia. Animal experiments showed that DM9218 could reduce serum UA level and hepatic xanthine oxidase activity and regulate intestinal dysbiosis induced by high fructose in fructose-fed mice (Wang et al., 2019). In addition, Garcia-Arroyo et al. demonstrated that probiotics containing urate-decomposing bacteria could reduce serum uric acid in hyperuricemic animals. It could also beneficially affect hypertension and kidney disease (Garcia-Arroyo et al., 2018).

A prebiotic published is “a substrate that is selectively utilized by host microorganisms conferring a health benefit (Swanson et al., 2020). Recent studies employing a variety of probiotic molecules have consistently demonstrated an increase in the relative numbers of *Lactobacillus* and *Bifidobacterium* spp. as well as changes in bacterial metabolism, as evidenced, in particular, by increased production of short-chain fatty acids, such as butyrate and propionate (Holscher, 2017). The research of prebiotics in treating gout has become a promising direction.

An animal experiment by Ren et al. found that fisetin reversed changes in *Bacteroides*, and *Firmicutes* in hyperuricemic mice, suggesting that fisetin reduced serum uric acid levels by modulating hyperuricemia-induced changes in gut microbiota. In addition, fisetin could improve renal function in hyperuricemia-induced CKD mice by regulating intestinal microbiota-mediated tryptophan metabolism (Ren et al., 2021). In addition, relevant metabolomics studies have shown that nuciferine may inhibit the pathological process of hyperuricemia by regulating the disturbed metabolic pathways. Furthermore, nuciferine can restore the metabolic changes caused by hyperuricemia by regulating intestinal microbiota composition in rats (Wang et al., 2020). E. prolifera polysaccharides (EPP), one of the most widely distributed green algae belonging to the family Ulvaceae, showed beneficial effects on the serum levels of UA and significantly improved the diversity of gut microbiota, especially the proportions of Alistipes and *Parasutterella*. Further, correlation analysis revealed that the presence of *Parasutterella* might be negatively associated with increased UA (Li et al., 2021).

In addition, it has been shown that co-feeding of β-carotene and green tea powder in gouty mice significantly increased the positive interaction between gut microbes, which may positively in relieve gout symptoms (Feng et al., 2022). Dietary administration of tuna meat oligopeptides (TMOP) alleviates hyperuricemia and renal inflammatory phenotypes. Furthermore, it reprograms the uric acid metabolism pathway. TMOP treatment repairs the intestinal epithelial barrier, reverses the dysbiosis of the gut microbiota, and increased the production of SCFAs. Furthermore, the antihyperuricemic effect of TMOP was transmitted by transplanting fecal microbiota from TMOP-treated mice, mediating the protective effect, at least in part, by the gut microbiota (Han et al., 2020).

In recent years, there have been more studies on the mechanism of various probiotics and prebiotics in treating gout. **Table 1** shows a summary of gout treatment targeting intestinal microorganisms. However, most studied conducted animal experiments, and no testing has been done on humans. Future research should focus more on human experiments to explore whether these new treatments, such as prebiotics and probiotics, can relieve the symptoms of gout and achieve the purpose of treatment.

**Table 1:** Mechanism of targeted gut microbiota in the treatment of gout.

	Type	Effect	Mechanism
Inulin (Guo et al., 2021)	Prebiotics	Reduces serum uric acid level, relieves inflammation and repairs intestinal epithelial barrier. Enhances microbial diversity and increases the relative abundance of beneficial bacteria.	Increase ABCG2 expression in the intestinal tract. Down-regulate XOD expression and activity in the liver of KO mice.
Chicory (Bian et al., 2020)	Prebiotics	Reduces serum uric acid level and increases fecal uric acid. Repairs intestinal mucosal injury.	Increases the number of probiotics and reduce the number of pathogenic bacteria to restore intestinal microbiota. Reduces the inflammatory response of the LPS/TLR4 axis by down-regulating the inflammatory pathways of serum LPS and TLR4/NF-κB in the kidney, thus promoting the excretion of uric acid in the kidney
Tuna meat oligopeptides (TMOP) (Han et al., 2020)	Prebiotics	Reduces hyperuricemia and renal inflammatory phenotype.	Reprograms the uric acid metabolic pathway to inhibit NLRP3 inflammasome activation and toll-like receptor 4/bone marrow differentiation factor 88/NF-kappaB (TLR4/MyD88/NF-κB) signal pathway, and the phosphorylation of p65 murine NF-κ B. Repairs the intestinal epithelial barrier. Reverses intestinal flora imbalance and increases short-chain fatty acids production.
Camellia japonica bee pollen polyphenols (CPE-E) (Xu et al., 2021)	Prebiotics	Reduces serum uric acid level and improve renal function.	Inhibits hepatic xanthine oxidase (XOD) activity and regulates the expression of URAT1, GLUT9, OAT1, OCT1 and ABCG2 in the kidney. Changes gut microbiota structure and increases the abundance of beneficial bacteria and the content of short-chain fatty acids. Decreases NLRP3 inflammasome and related inflammatory cytokines.
Sea cucumber hydrolysates (Wan et al., 2020)	Prebiotics	Reduces hyperuricemia and renal inflammation caused by diet.	Inhibits uric acid biosynthesis and promote uric acid excretion. Down-regulates pro-inflammatory cytokine transcription and up-regulates anti-inflammatory cytokine transcription. Inhibits TLR4/MyD88/NF-κB signal pathway. Increases the abundance of beneficial lactobacillus and short-chain fatty acids producers and reduces the abundance of opportunistic pathogens to alleviate intestinal microbiota dysfunction.
β-carotin and green tea powder (Feng et al., 2022)	Prebiotics	Relieves acute gout attack.	Reduces the joint swelling and pain in mice with gout. Reduces serum uric acid and pro-inflammatory cytokines levels. Improves the gut microbiota profile and reduces the metabolic levels of purines and pyrimidines.
Enteromorpha prolifera (Li et al., 2021)	Prebiotics	Reduces hyperuricemia and reverses kidney damage.	Decreases serum uric acid, serum urea nitrogen, serum xanthine oxidase (XOD), and hepatic XOD. Up-regulates UA excretion genes, such as ABCG2, OAT1, and NPT1. Down-regulates UA absorption gene URAT1 was down-regulated. Maintains intestinal flora stability, which is closely related to the regulation of hyperuricemia.
Hexapeptides derived from Apostichopus japonicus hydrolysate (Fan et al., 2022)	Prebiotics	Reduces uric acid biosynthesis and reabsorption.	Inhibits uric acid biosynthesis and reabsorption to reduce serum uric acid. Reduces renal inflammation and inhibits the activation of NLRP3 inflammasome. Decreases the richness and diversity of gut microbiota and changes the composition of phylum and genus levels. Changes miRNA expression in the kidney.
Anserine supplementation (Han et al., 2021)	Prebiotics	Promotes uric acid excretion. Has anti-inflammatory effects.	Increases hypoxanthine phosphate ribose transferase expression. Inhibits the uric acid synthesis by activating uric acid transporter. Inhibits NLRP3 inflammasome and TLR4/MyD88/NF-κB pathway. Regulates the composition and abundance of gut microbiota during hyperuricemia and renal inflammation.
Nuciferine (Wang et al., 2020)	Prebiotics	Relieves hyperuricemia and improves renal function.	Interferes with the gut microbiota and restores the metabolic balance of hyperuricemia rats. Reverses the levels of creatinine and creatine in rats to some extent after nuciferine treatment.
Fisetin (Ren et al., 2021)	Prebiotics	Prevents CKD induced by hyperuricemia.	Regulates tryptophan metabolism and aromatics receptor (AHR) activation mediated by gut microbiota.
Curcumin (Xu et al., 2021)	Prebiotics	Reduces the level of uremic toxin and improves renal inflammation and fibrosis.	Regulates gut microbiota’s structure and improves intestinal permeability. Increases beneficial bacteria and reduces pathogens.
AJOP (Lu et al., 2021)	Prebiotics	Relieves hyperuricemia.	Regulates uric acid metabolism, inhibits NLRP3 inflammasome and NF- κB signal pathway activation, and repairs intestinal epithelial barrier. Globally changes the spectrum of GIT microflora.
Lactobacillus brevis DM9218 (Wang et al., 2019)	Probiotics	Reduce serum uric acid level.	Down-regulates xanthine oxidase expression and activity stimulated by inflammatory cytokines.
Limosilacto-bacillus fermentum JL-3 (Wu et al., 2021)	Probiotics	Attenuates oxidative stress and inflammation induced by UA and regulates UA-induced flora imbalance in hyperuricemia mice.	Degrades UA in the intestine and reduces the amount of UA reabsorbed by intestinal epithelium into circulation. Contains purine-degrading lactobacillus strains and improves defecation activity, thus reducing fecal excretion of UA. Regulates gut microbiota’s structure and function.
Qu-Zhuo-Tong-Bi Decoction (QZTBD) (Wen et al., 2020)	Traditional Chinese medicine	Relieves acute gout attack.	Recovers the imbalance of gut microbiota and enhances SCFA formation. Inhibits intestinal barrier function, key glycolysis-related enzymes, and inflammatory factors production.
Fecal microbiota transplantation (Liu et al., 2020; Han et al., 2020; Xie et al., 2022)		Reduces serum uric acid level.	Restores damaged intestinal barrier function.

#### 5.2.2 Dietary habits

The microbiota composition can be modified by a variation in the individual’s dietary-nutritional habits, especially concerning the quality and quantity of fats, dietary fibers and carbohydrates consumed (Voreades et al., 2014). Dietary factors are also considered a risk factor for gout (Dehlin et al., 2020), thus, several well-established healthy eating patterns, such as the Mediterranean and Diet to Stop Hypertension (DASH) diets, can lower serum urate levels (Yokose et al., 2021). Cohort studies have shown that a typical western diet is associated with a higher risk of developing gout, while adherence to a Mediterranean diet is associated with a lower risk (Rai et al., 2017). Therefore, the Mediterranean and DASH diets have preventive effects on hyperuricemia (Sun et al., 2022).

In addition, studies have shown that an excessive fructose diet can affect gut microbiota composition through a series of damage to the intestinal barrier function of the inflammatory response. Therefore, a new method for gout treatment could be to limit the specific fructose intake and improve the composition of gut microbiota or targeted metabolite (Fang et al., 2022).

#### 5.2.3 Fecal microbiota transplantation

FMT is the transfer of fecal microbial content from a healthy individual into the gastrointestinal tract of a diseased individual (Ooijevaar et al., 2019). The action mechanism is not entirely understood, but restoring a disturbed microbiota underlies the observed effect (Smits et al., 2016). Since gut microbiota imbalance is closely related to gout, FMT may become a new direction for treating gout.

Xie et al. found that the washed microbiota transplantation (WMT) effectively reduced serum uric acid levels, relieved gout symptoms, and improved impaired intestinal barrier function in gout patients (Xie et al., 2022). In addition, a previous study showed that fecal transplantation attenuated hyperuricemia and renal inflammatory phenotypes in mice (Han et al., 2020).

#### 5.2.4 Traditional Chinese medicine

Traditional Chinese medicine (TCM) has been applied to treat gout since ancient China. Some chemical ingredients isolated from TCM herbs are multi-target and low toxicity, showing advantages and good prospects in gout prevention and treatment (Chi et al., 2020).

An animal study by Lin et al. demonstrated that Si Miao decoction improved gut microbiota dysbiosis associated with gouty arthritis by significantly reducing the abundance of *Prevotella* in the gut microbiota of mice, beneficial to relieve inflammation. In addition, some pathogenic bacteria positively correlated with intestinal inflammatory cytokines were reduced by Si Miao decoction, including *Klebsiella, Brautia, Escherichia-Shigella, and Enterococcus (* Lin et al., 2020).

Qu-Zhuo-Tong-Bi Decoction (QZTBD) has been shown to inhibit the growth of *Larrelidae* _A2, a bacterium enriched in gouty mice, and to improve the abundance of *ranunculus* (a bacterium closely related to SCFAs). QZTBD can exert its therapeutic effects by restoring the gut microbiota composition and SCFA production. QZTBD treatment attenuated intestinal mucosal barrier function and promoted intestinal uric acid excretion through these changes. Furthermore, it inhibited glycolysis and suppressed intestinal proinflammatory cytokines (Wen et al., 2020). Therefore, traditional Chinese medicine targeting intestinal flora in gout treatment may be a promising direction

## 6 Conclusions

Gut microbiota plays a key role in gout pathogenesis through the changes of diversity, abundance, metabolic pathway, and metabolites, such as SCFAs, resulting in hyperuricemia and gout flare. Hyperuricemia and gout can cause an imbalance in the microbiota in the gut, which can then trigger the development of other metabolic illnesses, creating a vicious cycle. In addition, drugs related to gout treatment can play a therapeutic role by changing the composition of gut microbiota. Gut microbiota examination provides a non-invasive, simple, sensitive, and reliable index in diagnosis. Developing novel and safe new drugs targeted at gut microbiota has become a research focus. Prebiotics, probiotics, traditional Chinese medicine and fecal transplantation therapy are expected to become new methods for gout treatment.

## Author contributions

ST: Writing - Original Draft. PZ and MC: Visualization. QC and XC: Resources. ZW: Methodology. XL: Writing- Reviewing and Editing. HW: Conceptualization. All authors contributed to the article and approved the submitted version.

## Funding

This work was supported by the Key Research and Development Program of Zhejiang Province (No. 2020C3044) and the National Natural Science Foundation of China (No. 82071810).

## Conflict of interest

The authors declare that the research was conducted in the absence of any commercial or financial relationships that could be construed as a potential conflict of interest.

## Publisher’s note

All claims expressed in this article are solely those of the authors and do not necessarily represent those of their affiliated organizations, or those of the publisher, the editors and the reviewers. Any product that may be evaluated in this article, or claim that may be made by its manufacturer, is not guaranteed or endorsed by the publisher.
